# Decreased expression of hyaluronan synthase 1 and 2 associates with poor prognosis in cutaneous melanoma

**DOI:** 10.1186/s12885-016-2344-8

**Published:** 2016-05-16

**Authors:** Mari Poukka, Andrey Bykachev, Hanna Siiskonen, Kristiina Tyynelä-Korhonen, Päivi Auvinen, Sanna Pasonen-Seppänen, Reijo Sironen

**Affiliations:** Institute of Biomedicine, University of Eastern Finland, P.O. Box 1627, 70211 Kuopio, Finland; Cancer Center, Kuopio University Hospital, Kuopio, Finland; Department of Dermatology, University of Eastern Finland and Kuopio University Hospital, Kuopio, Finland; Institute of Clinical Medicine/Clinical Pathology, University of Eastern Finland, Kuopio, Finland; Department of Clinical Pathology, Kuopio University Hospital, Kuopio, Finland; Cancer Center of Eastern Finland, Kuopio, Finland

**Keywords:** Hyaluronan, Melanoma, Hyaluronan synthases 1 and 2, Hyaluronidase 2, Prognosis, Lymph node metastasis

## Abstract

**Background:**

Hyaluronan is a large extracellular matrix molecule involved in several biological processes such as proliferation, migration and invasion. In many cancers, hyaluronan synthesis is altered, which implicates disease progression and metastatic potential. We have previously shown that synthesis of hyaluronan and expression of its synthases 1–2 (HAS1-2) decrease in cutaneous melanoma, compared to benign melanocytic lesions.

**Methods:**

In the present study, we compared immunohistological staining results of HAS1 and HAS2 with clinical and histopathological parameters to investigate whether HAS1 or HAS2 has prognostic value in cutaneous melanoma. The specimens consisted of 129 tissue samples including superficial (Breslow ≤ 1 mm) and deep (Breslow > 4 mm) melanomas and lymph node metastases. The differences in immunostainings were analysed with non-parametric Mann–Whitney U test. Associations between immunohistological staining results and clinical parameters were determined with the χ^2^ test. Survival between patient groups was compared by the Kaplan-Meier method using log rank test and Cox’s regression model was used for multivariate analyses.

**Results:**

The expression of HAS1 and HAS2 was decreased in deep melanomas and metastases compared to superficial melanomas. Decreased immunostaining of HAS2 in melanoma cells was significantly associated with several known unfavourable histopathologic prognostic markers like increased mitotic count, absence of tumor infiltrating lymphocytes and the nodular subtype. Furthermore, reduced HAS1 and HAS2 immunostaining in the melanoma cells was associated with increased recurrence of melanoma (*p* = 0.041 and *p* = 0.006, respectively) and shortened disease- specific survival (*p* = 0.013 and *p* = 0.001, respectively).

**Conclusions:**

This study indicates that reduced expression of HAS1 and HAS2 is associated with melanoma progression and suggests that HAS1 and HAS2 have a prognostic significance in cutaneous melanoma.

**Electronic supplementary material:**

The online version of this article (doi:10.1186/s12885-016-2344-8) contains supplementary material, which is available to authorized users.

## Background

Cutaneous melanoma is an aggressive type of skin cancer originating from pigment-producing melanocytic skin cells. The incidence of cutaneous melanoma among fair-skinned populations has risen significantly in recent decades [1, 2]. The main risk factors for melanoma are ultraviolet (UV) exposure and the presence of melanocytic nevi [3, 4]. In the early stages of the disease, cutaneous melanoma is curable with surgical excision. However, as the disease progresses, melanoma cells acquire the ability to metastasize. Cutaneous melanoma is highly metastatic, and even in the early phases of the disease there is a small subgroup of thin melanomas that develop metastases and are not able to be cured surgically. Unfortunately, there are no accurate prognostic or diagnostic biomarkers currently available to predict the progression of this disease.

Hyaluronan is a large glycosaminoglycan residing in the extracellular matrix of most human tissues. It is expressed abundantly in normal skin, in both the epidermis and dermis. Hyaluronan is formed on the plasma membrane by three hyaluronan synthases (HAS 1–3) and during its synthesis it is discharged into the extracellular matrix. Hyaluronan, together with its primary cell surface receptor CD44, have been shown to contribute to processes necessary for cancer development such as migration, invasion and resistance to chemotherapeutic drugs [5–9]. In addition, it has been reported that hyaluronan and hyaluronan-fragments have angiogenic properties in human endothelial cells and hyaluronan contributes to wound healing and leukocyte adhesion via long hyaluronan cables [10–12].

The role of hyaluronan in melanomagenesis has remained obscure, partly due to previously published conflicting results. Some in vitro studies suggest that hyaluronan promotes melanoma cell migration and invasion [13, 14], while in vivo studies indicate that reduced expression of hyaluronan correlates positively with the invasiveness of cutaneous melanoma [15, 16]. In mouse models, elevated levels of circulating hyaluronan have been shown to associate with decreased lung metastases [17]. The expression of CD44 and hyaluronan is decreased in human cutaneous melanomas and this is associated with the progression of disease and poor prognosis [15]. Our previous work showed that hyaluronan content is increased in the in situ melanomas compared to benign nevi, whereas deep melanomas (Breslow > 4 mm) are almost devoid of hyaluronan [16]. Similar decreased hyaluronan content has been shown in squamous cell carcinomas (SCCs) of larynx, mouth and skin, which are tumors originating from stratified epithelia [18–20]. Decreased tumoral hyaluronan content is accompanied by an increase in the hyaluronan degrading enzyme, hyaluronidase 2 (HYAL2), and a decrease in HAS1 and HAS2 expression in invasive melanomas and lymph node metastases compared to benign nevi and in situ melanomas [16]. In contrast, hyaluronan content seems to be increased in tumors originating from simple epithelia [7]. Thus, adenocarcinomas of the breast, colorectal and ovary have abundantly hyaluronan in the tumor and stromal cells and this correlates with an unfavorable prognosis [21–23].

Our previous work showed that decreased expression of hyaluronan in the cutaneous melanoma is due to decreased expression of HAS1 and HAS2 and increased expression of HYAL2. In the present study our aim was to investigate whether HAS1-2 or HYAL2 have prognostic value for cutaneous melanoma. Here we demonstrate for the first time that decreased expression of HAS1 and HAS2 favours melanoma progression and metastasis. The immunostaining of both HAS1 and HAS2 was decreased in deeply invasive melanomas and lymph node metastases compared to superficial melanomas and this associated with several known negative prognostic factors. These tumors showed high HYAL2 immunostaining levels but interestingly, it did not affect prognosis of patients. Our work delivers new information about hyaluronan metabolism in cutaneous melanoma and identifies HAS1 and HAS2 as possible prognostic factors in this aggressive cancer.

## Methods

### Histological samples and clinical data

Paraffin embedded diagnostic tissue samples were taken from invasive cutaneous melanomas (thickness < 1 mm or > 4 mm, *n* = 82) and lymph node metastases (*n* = 47) diagnosed between 1980–2010 in Kuopio University Hospital. Invasive melanomas with Breslow depths less than 1 mm or more than 4 mm were chosen to investigate the difference between the groups representing relatively different survival in general (in melanomas < 1 mm the 10-year survival is about 92 %, while in melanomas > 4 mm it is only about 10 %). The histopathological parameters were re-evaluated by an experienced histopathologist (R.S), and the clinical patient data was collected. The research has been approved by Committee on Research Ethichs of the North Savo Hospital District and The Finnish National Supervisory Authority for Welfare and Health (VALVIRA). The registry study protocol was retrospective and thus the consent of the patients for participation was not required.

### HAS1 and 2 and HYAL2 immunohistological stainings

After deparaffinization, the tissue sections were cooked in 10 mM citrate buffer (pH 6.0) in a pressure cooker for 15 minutes and after cooling washed with 0.1 M phosphate buffer (PB; pH 7.0). The endogenous peroxidase activity was blocked with 1 % H_2_O_2_ for 5 minutes. Thereafter the sections were washed and incubated with 1 % bovine serum albumin (BSA), 0.05 % Tween-20 and 0.1 % Gelatin (Sigma G-2500) in PB for 30 minutes at 37 °C to block unspecific binding. After blocking, the sections were incubated with goat polyclonal antibodies for hyaluronan synthases diluted in 1 % BSA (HAS1 antibody 1:100 dilution and HAS2 antibody 1:120 dilution, Santa Cruz Biotechnology, Santa Cruz, CA). HYAL2 2 was stained with rabbit polyclonal antibody, (1:100 Abcam, Cambridge, UK). In controls, the primary antibody was omitted. The specificity of the HAS and HYAL2 antibodies was tested as described in our previous work [16]. The sections were incubated at 4 °C overnight with primary antibodies. The following day, the sections were rinsed with PB and incubated with biotinylated secondary antibodies, anti-goat antibody (1:1000, Vector Laboratories) diluted with 1 % BSA in PB for HASes and anti-rabbit antibody (1:200, Vector Laboratories) for HYAL2. The bound antibodies were visualized with avidin-biotin-peroxidase method (1:200, Vector Laboratories, Irvine, CA) using 0.05 % 3,3-diaminobenzidine (DAB, Sigma, St.Louis, MO) as a substrate. The Mayer’s hematoxylin counterstained sections were mounted in DePex (BDH Laboratory Supplies, Poole, England).

### Evaluation of immunohistological stainings

The evaluation of the immunostainings was done independently by two researchers (M.P., H.S.). The immunostaining coverages and the intensities were evaluated in melanoma and stromal cells as previously described [16]. The amounts of immunopositive cells were estimated with a five-level scoring system as follows; 1 = 0-5 %, 2 = 6-25 %, 3 = 26-50 %, 4 = 51-75 %, 5 = 76-100 % (Additional file 1: Figure S1). The intensities of the immunostainings were estimated with a four-level scoring system from 0 to 3 as follows; negative (0), weak (1), moderate (2) or strong (3).

### Statistical analyses

Statistical analyses were performed with SPSS Statistics 21 (IBM). The differences in immunostainings between all stages (pT1, pT4 and pN1-) were analysed with non-parametric Mann–Whitney U test. Associations between immunostainings and clinical data were determined with χ2 test. For the χ2 test continuous variables were transformed into categorical variables. Univariate survival analyses of different groups were determined with Kaplan-Meier log rank test. Two Kaplan-Meier log rank test were performed to verify the accuracy of clinical data (Additional file 2: Figure S2). Multivariate analyses were performed with the Cox regression model. The multivariate analyses tests were conducted separately for two different groups because some histopathological covariates were only analysed from primary cutaneous melanoma samples (pT1 and pT4). Tests were conducted only for primary cutaneous melanomas (pT1 and pT4) without lymph node metastasis and for all stages (pT1, pT4 and pN1-). The immunostaining categories 0 (0-5 %) and 1 (6- 26 %) were merged in the χ2 test, Kaplan-Meier log rank test and Cox regression model because of small group sizes. P-values less than 0.05 were considered statistically significant.

## Results

Clinical information and histological samples were obtained from 129 patients; 74 males (57.4 %) and 55 (42.6 %) females (Table 1). The samples consisted of 41 superficial melanomas (Breslow ≤ 1 mm, pT1), 41 deep melanomas (Breslow > 4 mm, pT4) and 47 lymph node metastases of melanoma (pN1). The most common cutaneous localization of primary melanoma was the back (26.4 %). The mean age at the time of diagnosis was 59 (ranging between 5 – 92 years) and the mean follow-up time was 8.2 years (ranging between 0.1 – 32.67 years). 71 (55.0 %) patients had relapse or widely metastatic disease at the time of diagnosis (Table 1). Interferon treatment, chemotherapy and radiation therapy was given to 32 (24.8 %), 36 (27.9 %) and to 41 (31.8 %) patients, respectively, with metastatic disease (data not shown).

**Table 1:** Clinical information of the patients (*n* =129)

Variable	pT1	pT4	pN1-	Total
Number of cases	41 (31.8 %)	41 (31.8 %)	47 (36.4 %)	129 (100.0 %)
Gender				
Male	22 (17.1 %)	24 (18.6 %)	28 (21.7 %)	74 (57.4 %)
Female	19 (14.7 %)	17 (13.2 %)	19 (14.7 %)	55 (42.6 %)
Age				
under 20	0 (0.0 %)	1 (0.8 %)	1 (0.8 %)	2 (1.6 %)
20-59	18 (14.0 %)	14 (10.9 %)	26 (20.2 %)	58 (45.0 %)
60 and over	23 (17.8 %)	26 (20.2 %)	20 (15.5 %)	69 (53.5 %)
Any relapse				
Yes	3 (2.4 %)	23 (18.1 %)	39 (30.7 %)	65 (51.2 %)
No	37 (29.1 %)	16 (12.6 %)	3 (2.4 %)	56 (44.1 %)
Spread at diagnosis	0 (0.0 %)	1 (0.8 %)	5 (3.9 %)	6 (4.7 %)
Alive				
Yes	31 (24.0 %)	8 (6.2 %)	9 (7.0 %)	48 (37.2 %)
No	10 (7.8 %)	33 (25.6 %)	38 (29.5 %)	81 (62.8 %)
Cause of death				
Melanoma	1 (1.2 %)	20 (24.7 %)	32 (39.2 %)	53 (65.4 %)
Other	5 (6.2 %)	7 (8.6 %)	0 (0.0 %)	12 (14.8 %)
Not known	4 (4.9 %)	6 (7.4 %)	6 (7.4 %)	16 (19.8 %)

### Decreased expression of HAS 1 and HAS 2 is associated with the more advanced stages of melanoma

**Fig. 1:**
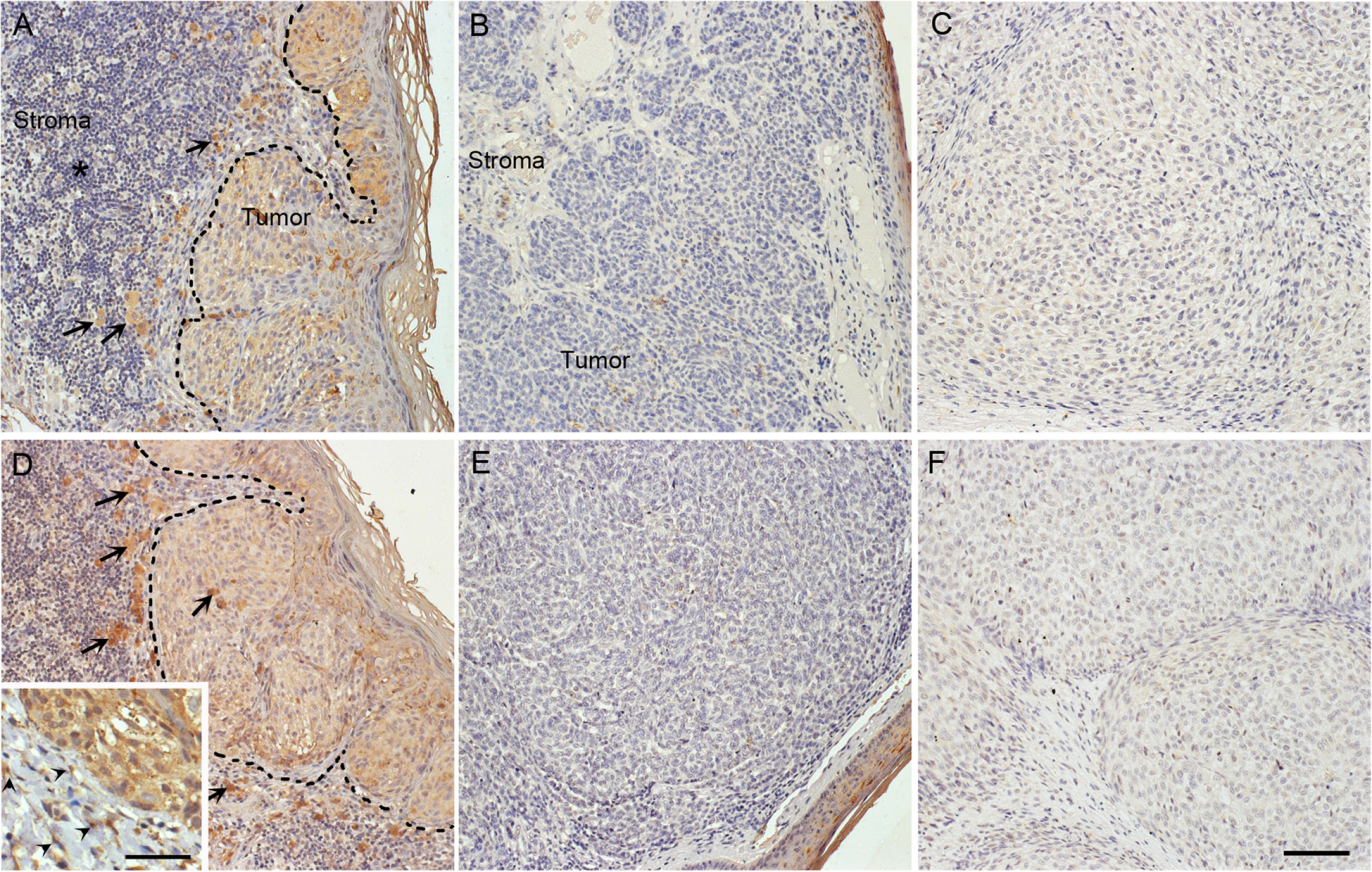
HAS1 and HAS2 immunoreactivity in superficially and deeply invasive melanomas and in lymph node metastases. Immunostainings of HAS1 **a**-**c** and HAS2 **d**-**f** in superficially **a** and **d** and deeply invasive melanomas **b** and **e** and in lymph node metastases **c** and **f**. Black dash lines in **a** and **d** mark the border between the tumor and the stroma. Black asterisk in **a** points to numerous tumor infiltrating lymphocytes in superficial melanoma. Black arrows **a**, **d** indicate melanin containing tumor cells in superficial melanoma and black arrowheads in (D, insert) point to HAS2 immunopositive stromal cells. In deep melanoma and lymph node metastasis tumor cells show weak immunostaining or are totally negative **b**, **c**, **e**, **f**. Scale bars 100 μm

In superficial melanoma, melanoma cells were diffusely immunostained with both HASes (Fig. 1a, d). In melanoma cells, both the cytoplasm and plasma membrane showed immunoreactivity (up to 90 %), whereas most (up to 80 %) of the stromal cells showed no immunopositivity (Additional file 3: Figure S3). When expressed, HAS1 and HAS2 were localized in the cytoplasm and on the plasma membrane of stromal cells (Fig. 1d, insert).

**Fig. 2:**
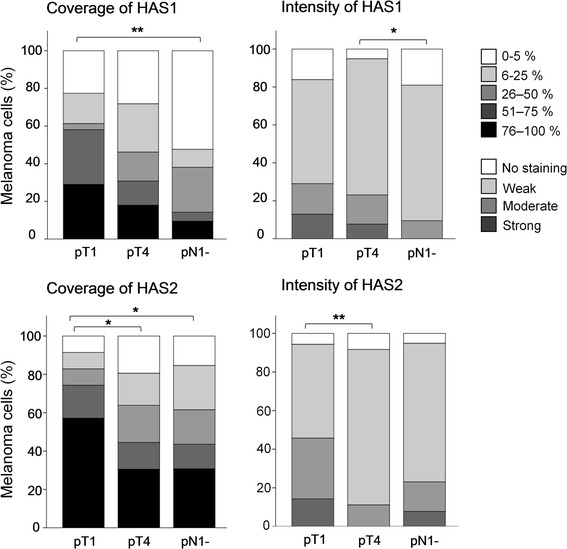
Coverage and intensity of HAS1 and HAS2 immunostainings in melanoma cells of superficial melanoma (pT1), deep melanoma (pT4) and lymph node metastasis (pN1-). Coverage and intensity of HAS1 immunostainings were successfully recorded from 112 samples. Coverage and intensity of HAS2 immunostainings were recorded from 110 samples. Statistically significant differences between the stages are indicated with brackets (Mann–Whitney U test). * *p*-value < 0.05, ** *p*-value <0.01, *** *p*-value <0.001

A decrease in HAS1 positive melanoma cells was associated with advanced stage melanoma (*p* = 0.006; Table 2 and Fig. 2). Thus, the proportion of HAS1 immunopositive melanoma cells was significantly lower in LN metastases than in superficial (pT1) melanomas (*p* = 0.002; Fig. 2). Similarly, the proportion of HAS2 immunopositive melanoma cells was significantly lower in deeply invasive (pT4) melanomas and LN metastases (pN1) (*p* = 0.013 and *p* = 0.012, respectively) compared to superficial melanomas (Fig. 2). In addition, staining intensity of HAS1 in melanoma cells was decreased in LN metastases compared to deeply invasive melanomas (*p* = 0.018, Fig. 2) and HAS2 intensity in melanoma cells was decreased in deeply invasive melanomas compared to superficial ones (*p* = 0.002; Fig. 2). Decreased HAS2 intensity in melanoma cells was also associated with advanced stage (*p* = 0.047; Table 2).

**Table 2:** Correlation of HAS1 and HAS2 with clinical and histopathological factors

Variable	HAS1 Coverage	HAS1 Intensity	HAS2 Coverage	HAS2 Intensity
Stage	*p* = 0.006	ns	ns	*p* = 0.047
pT1, pT4 or pN1-				
Melanoma related death	*p* = 0.007	ns	*p* = 0.001	*p* = 0.016
Recurrence	*p* = 0.021	ns	*p* = 0.007	ns
Regional recurrence	*p* = 0.006	p = 0.023	*p* = 0.007	ns
Distant recurrence	*p* = 0.012	ns	*p* = 0.001	*p* = 0.004
Genre	ns	ns	ns	ns
Age	ns	ns	ns	ns
Ulceration	ns	ns	ns	ns
TIL (low / moderate / high)	ns	ns	*p* = 0.036	*p* = 0.040
Mitotic count (mitosis/mm^2^)	ns	ns	ns	*p* = 0.018
Horizontal diameter of melanoma (mm)	ns	ns	*p* = 0.002	*p* =0.042
Growth type				
Nodular	ns	ns	ns	*p* = 0.001

Stage = pT1, pT4 or pN1-. TIL = tumor-infiltrating lymphocytes (evaluated either low, moderate or high amount). Mitosis = mitosis/mm2, horizontal tumor diameter (mm)

The overall proportion of immunopositive stromal cells was 0-5 % (Additional file 3: Figure S3). Similar to melanoma cells, the strongest HAS1 and HAS2 immunostaining intensity in stromal cells was observed in superficial melanomas (Fig. 1). Decreased immunostaining intensity was observed in LN metastases compared with superficial melanomas (p = 0.013 for HAS1, p < 0.001 for HAS2, Additional file 3: Figure S3).

### Low HAS1 and HAS 2 expression is associated with melanoma related death

Decreased coverage of HAS2 immunostaining in melanoma cells was associated with several histopathological factors, including reduced number of tumor infiltrating lymphocytes (TILs) (*p* = 0.036) and increased horizontal tumor diameter (*p* = 0.002; Table 2). Results were similar for the intensity of HAS2 immunostaining; lower HAS2 intensity was associated with a reduced number of TILs (*p* = 0.040), a larger horizontal diameter (*p* = 0.042), nodular subtype (*p* = 0.001) and an increased mitotic activity (*p* = 0.018; Table 2). On the other hand, increased intensity of HAS2 in melanoma cells was associated with superficial type (*p* = 0.047; data not shown). Neither coverage nor intensity of HAS1 staining associated with any histopathological factors (Table 2). Reduced HAS2 immunostaining (coverage and intensity) was associated with melanoma-related death (*p* = 0.001 and *p* = 0.016, respectively; Table 2). Furthermore, decreased HAS1 coverage in melanoma cells (*p* = 0.007; Table 2), and decreased intensity of HAS2 in the stromal cells, was positively associated with melanoma-related death (*p* = 0.038; data not shown). Reduced coverage of HAS1 and HAS2 in melanoma cells was associated with recurrence of the disease, both regional and distant (*p* = 0.021 and *p* = 0.007, respectively; Table 2 recurrence). Increased regional recurrence was related to reduced number of HAS1 and HAS2 –positive melanoma cells (*p* = 0.006 and *p* = 0.007, respectively; Table 2 regional and distant recurrence). Similarly, increased distant recurrence was related to reduced HAS1 and HAS2 positive melanoma cells (*p* = 0.012 and *p* = 0.001, respectively; Table 2). Decreased intensity of HAS1 in melanoma cells was related to increased regional metastasis (*p* = 0.023; Table 2), while decreased intensity of HAS2 was associated with distant metastasis (*p* = 0.004; Table 2).

### Reduced expression of HAS 1 and HAS 2 is associated with decreased disease-specific survival

**Fig. 3:**
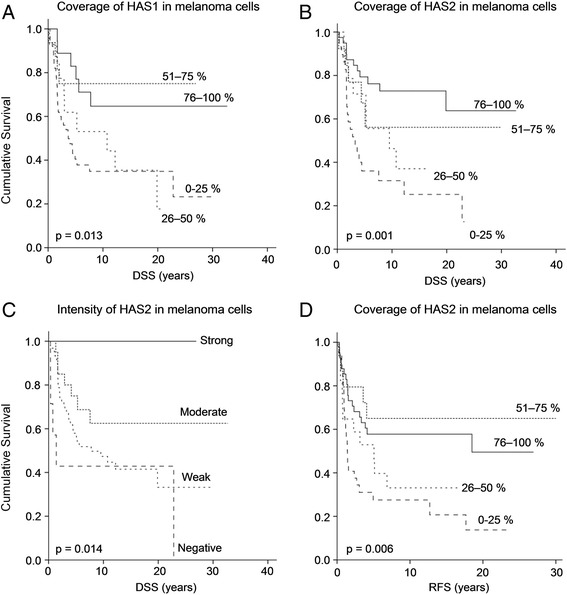
Kaplan-Meier survival curves according to HAS1 and HAS2 expression. Kaplan-Meier log rank test indicating association of decreased HAS1 **a** and HAS2 **b** coverage and decreased intensity of HAS2 **c** in melanoma cells with declined disease-specific survival. Kaplan-Meier log rank test indicating association of decreased HAS2 coverage with declined recurrence-free survival **d**. DSS = disease-specific survival, RFS = recurrence-free survival

At the end of the follow-up time, 48 patients were alive and 81 had deceased. In melanoma cells, a reduced amount of HAS1 positivity was associated with decreased disease-specific survival (DSS) (*p* = 0.013; Fig. 3) and recurrence-free survival (RFS) (*p* = 0.041, data not shown). Similarly, decreased HAS2 coverage in melanoma cells was associated with poorer DSS (*p* = 0.001; Fig. 3) and RFS (*p* = 0.006; Fig. 3), and decreased intensity of HAS2 staining was related to shortened DSS (*p* = 0.014; Fig. 3). In contrast, HAS1 intensity in melanoma cells was not associated with DSS. In stromal cells, HAS1 staining was not associated with either DSS or RFS, while decreased intensity of HAS2 was associated with poorer DSS (*p* = 0.049, data not shown) and RFS (*p* = 0.008, data not shown).

Multivariate analyses were done in two different ways; for the primary cutaneous melanomas only (pT1 and pT4) and for all stages (pT1, pT4 and pN1). Covariates used in cutaneous melanomas (pT1 and pT4) were: Breslow’s classification, ulceration, mitotic rate, patients age and immunostaining results of HAS1 and HAS2. Significant adverse prognostic factors for decreased DSS were increased Breslow’s depth (*p* = 0.001) and decreased HAS1 and HAS2 staining intensity in melanoma cells (*p* = 0.019 and *p* = 0.011, respectively). For RFS, significant adverse prognostic factors were deep invasion (*p* < 0.001) and decreased HAS2 staining intensity of melanoma cells (*p* = 0.014).

Covariates used in multivariate analyses for all stages (pT1, pT4 and pN1) were: patients’ age, stage (pT1, pT4 and pN1) and immunostaining results of HAS1 and HAS2. Decreased coverage of HAS2 positive melanoma cells was a significant negative prognostic factor (*p* = 0.039) for DSS, similar to increased stage (*p* = 0.001) and age (*p* = 0.035). HAS1 immunostaining did not have prognostic value for DSS.

### Expression of Hyaluronidase 2 in melanoma

**Fig. 4:**
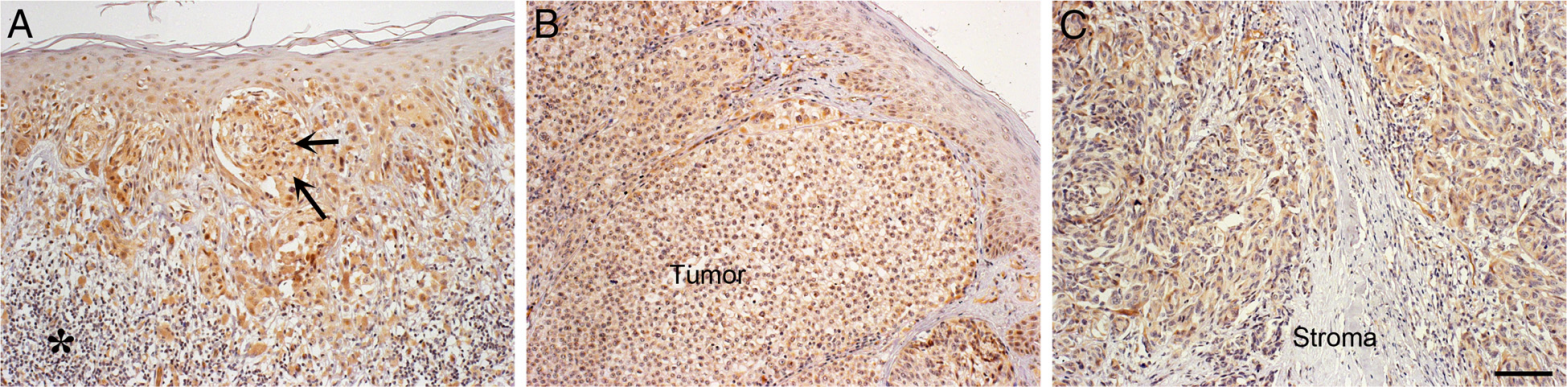
HYAL2 immunostaining in superficially **a** and deeply invasive melanomas **b** and in lymph node metastases **c**. Black asterisk in **a** points to numerous tumor infiltrating lymphocytes and black arrows immunopositive tumor cells in superficial melanoma. Scale bar 100 μm

HYAL2 immunostaining localized mostly on the cytoplasm of the melanoma cells (Fig. 4). The proportion of HYAL2 positive melanoma cells was mostly high (76–100 %, data not shown) for all stages. Immunostaining intensities of HYAL2 were statistically uniform in all stages (Fig. 4). Between 50–60 % of samples had weak intensity in all stages (data not shown).

Coverage of stromal immunostaining was between 0–50 % with no statistical differences between stages, and there were no differences in the intensities of stromal staining. In all stages, the staining intensities of stromal cells were either weak or there were no staining.

## Discussion

The present work demonstrates that reduced expression of HAS1 and HAS2 is associated with an unfavorable prognosis in cutaneous melanoma. Reduced expression of HAS1 and HAS2 is significantly associated with reduced DSS and RFS. In addition, weak immunostaining of HAS2 in melanoma cells is associated with unfavorable histopathologic prognostic markers such as increased mitotic count, absence of tumor infiltrating lymphocytes and nodular subtype. Furthermore, multivariate analysis indicates that decreased expression of HAS1 and HAS2 in melanoma cells are independent prognostic factors.

Decreased tumoral hyaluronan content has been shown to be an adverse prognostic factor in cutaneous melanoma [15]. We have previously demonstrated that decreased expression of HAS1 and HAS2 and increased expression of hyaluronan degrading enzyme HYAL2 correlates with decreased tumoral hyaluronan content in the invasive melanomas [16]. In the present work, we showed that decreased expression of HAS1 and HAS2 are adverse prognostic factors, while the expression of HYAL2 does not affect the prognosis. Previously we showed that HYAL2 expression is elevated in dysplastic nevi and the expression is also elevated in locally invasive and metastatic melanomas [16]. Whereas the expression of HAS1 and HAS2 correlated with the content of hyaluronan in tumor tissue, and their expressions are not altered until the invasive phase of the disease, at which time hyaluronan content decreased [16]. This suggests that HASes are responsible for intratumoral hyaluronan concentration and they may have an adverse impact on tumor progression by modulating hyaluronan content in the tumor tissue.

Several melanoma cell lines synthesize substantial amounts of hyaluronan in vitro [24, 25]. Furthermore, melanoma cell-derived factors are able to induce hyaluronan synthesis in cutaneous fibroblasts via upregulation of HAS2 [26]. These findings suggest that in melanoma hyaluronan is produced by both melanoma and stromal cells, but most likely the majority of intratumor hyaluronan originates from melanoma cells. Our previous work included in situ melanomas, which expressed excessively hyaluronan [16]. Since in situ melanomas localize in epidermis without any proper stromal component, it is possible that the most of hyaluronan in these tumors originates from melanoma cells and also from epidermal keratinocytes, which are known to express all HASes [27]. In addition to hyaluronan, several other extracellular matrix molecules are also shown to be involved in melanomagenesis like versican and fibronectin [28–30]. Silencing of versican increases cell proliferation and migration, whereas silencing of fibronectin increases drug sensitivity of melanoma cells [28, 30].

Hyaluronan metabolism in cutaneous melanoma seems to differ from the main adenocarcinomas, such as the breast carcinoma. Increased stromal hyaluronan content has been associated with poor survival and tumor differentiation in various human adenocarcinomas, whereas reduced levels of hyaluronan are associated with worsened survival in melanoma and squamous cell carcinomas (SCC) of the larynx, mouth and skin [15, 18–20]. Normal skin, both epidermis and dermis, contains extensive amounts of hyaluronan. Interestingly, the hyaluronan concentration is further increased in the in situ phase of melanoma [16]. This increase in the early phase lesions has also been observed in cutaneous, laryngeal and oral SCCs [16, 18, 19, 31]. The results suggest that loss of hyaluronan is associated with the acquisition of a motile, invasive tumor cell phenotype. Increased content of hyaluronan may reflect an attempt to maintain hyaluronan synthesis at levels that are normal for the respective tissues. Physiologically, hyaluronan acts as a protective barrier against harmful substances, microbes and UV radiation. Rauhala showed that UVB exposure in keratinocytes causes increased hyaluronan synthesis via up-regulation of HAS1-3, which may have a protective effect on cells by increasing viability and decreasing the secretion of inflammatory mediators [27, 32]. Decrease of hyaluronan content in invasive melanoma and SCCs may increase invasiveness of the tumor cells, which is in agreement with recent findings where the accumulation of high molecular mass hyaluronan exerted anticancer like effects in naked mole rats [33]. This phenomenon was related to the exceptionally large molecular size of hyaluronan in these animals. Indeed, activation of hyaluronan degrading enzyme, HYAL2, led to a reduction in the high molecular mass hyaluronan, which resulted in tumor promotion in this model [33]. Moreover, our unpublished in vitro observations support the idea that hyaluronan overexpression tends to restrict melanoma cell growth, and melanoma cell lines (MV3 and C8161) overexpressing HAS3 show reduced cell motility and proliferation [25].

Knowledge of the prognostic significance of hyaluronan synthases in malignant tumors is currently relatively limited. In contrast to melanoma, increased HAS1-3 immunoreactivity is associated with poor survival in breast cancer [34]. In particular, HAS2 has been shown to suppress tissue metalloproteinase inhibitor 1 which increases the invasiveness of breast cancer cells [35]. Furthermore, increased transcription levels of HAS1 and HYAL1 are associated with metastasizing urothelial bladder carcinoma [36]. The prognostic value of a reduced HAS1-2 expression likely comes from decreased synthesis of hyaluronan. However, the finding that HAS1 and 2 are independent prognostic factors in melanoma raises the possibility that these enzymes by themselves affect tumor progression. For example, our unpublished in vitro observations indicate that cell adhesion is reduced in melanoma cells overexpressing HAS3 and this ability are not reversed with eradication of hyaluronan [25]. These results suggest hyaluronan synthesizing enzymes may independently affect cell function in the absence of any direct effects on hyaluronan synthesis.

Our results demonstrate that the proportion of HAS1 and HAS2 and hyaluronan positive melanoma cells is significantly decreased in lymph node metastases, compared with superficially invasive melanoma. These results indicate that decreased expression of HAS1 and HAS2, and thus reduced tumoral hyaluronan content, is a favorable feature for metastatic melanoma cells. Similarly, ovarian carcinoma cells synthesizing low amounts of hyaluronan were most adherent to the intra-abdominal peritoneal surfaces, suggesting that a large pericellular hyaluronan coat acts as a barrier for adhesion and inhibits peritoneal dissemination [37]. In addition to HAS1 and HAS2 expression, the observed HYAL2 expression may contribute to melanoma progression. The presence of hyaluronidase and hyaluronan fragments produced by hyaluronidases has been shown to mediate tumor progression by stimulating angiogenesis and tumor invasion [38, 39]. HYAL2 degrades hyaluronan to oligosaccharides, which may induce cleavage of the main cell surface hyaluronan receptor, CD44, resulting in increased motility and invasion [40]. HYAL2 has also been shown to directly cleave CD44, which may disturb the hyaluronan-CD44 interaction and release locally growing melanoma cells enabling the cells to spread [41]. In addition to CD44 shedding, the expression of certain CD44 variants has been shown to induce disease dissemination [42, 43]. Indeed, the expression of receptor variant CD44v6 associates strongly with brain metastases [42].

Our results indicate that reduced immunopositivity of HAS2 is associated with several known unfavorable histopathologic prognostic markers like a reduced number of tumor infiltrating lymphocytes (TIL), an increased melanoma horizontal diameter, an increased mitotic activity and the nodular subtype. The proinflammatory effect of hyaluronan has been previously comprehensively demonstrated [10, 44, 45]. The presence of hyaluronan depositions, and the formation of hyaluronan cables, recruits leukocytes to the site of inflammation and leukocytes binding to these cables occur mainly via CD44 [46, 47]. Interaction of hyaluronan-CD44 is important in numerous inflammatory diseases, such as allergic dermatitis and inflammatory liver disease [48–50]. In melanoma, hyaluronan may increase leukocyte infiltration, and therefore, the loss of hyaluronan could contribute to a reduction in TILs, thereby worsening the prognosis [51, 52]. This is an interesting finding since the majority of new therapies in metastatic melanoma operate through activation of immune responses [53].

## Conclusions

Caught in its early stages, melanoma can be cured by surgery. However, despite a recent surge in the development of new targeted therapies, metastatic melanoma remains a major challenge to treat. Our novel data provides novel information about hyaluronan metabolism in cutaneous melanoma and points towards a significant role for HAS1 and HAS2 in melanoma dissemination. Our results about correlation between decreased immunostaining of HAS1 and HAS2 and decreased survival of patients support the previous works and bring us new information about histopathological changes that happen during melanoma progression. However, whether decreased expression of HAS1 and HAS2 is the cause or a secondary consequence of cutaneous melanoma is a question that awaits further investigation.

## Additional files

Additional file 1: Figure S1. Evaluation of immunopositivity using five-level scoring system. **a** and **b** representing 0-5 % of melanoma cells stained positively, **c** representing 6-25 % of melanoma cells stained positively, **d** representing 26-50 % of melanoma cells stained positively, **e** representing 51-75 % melanoma cells stained positively and **f** representing 76-100 % of melanoma cells stained positively. Scale bar 100 μm. (TIF 19623 kb) Additional file 2: Figure S2. Clinical data’s accuracy was verified with two Kaplan-Meier log rank tests. Kaplan-Meier log rank test according stage (pT1, pT4 and pN1-) **a**. Patients with pT1 melanoma had better prognosis than patients with pT4 melanoma or lymph node metastasis (*p* <0.001). Kaplan-Meier log rank test according tumor-infiltrating lymphocytes –status **b**. Tumor infiltrating lymphocytes status of pT1 and pT4 melanomas were analyzed and higher amounts of tumor-infiltrating lymphocytes associated with better prognosis (*p* = 0.006). DSS = disease-specific survival. (TIF 536 kb) Additional file 3: Figure S3. Immunostaining results of HAS1 and HAS2 in stromal cells. Coverage and intensity of HAS1 and HAS2 immunostainings in the stroma of superficial melanoma (pT1), deep melanoma (pT4) and lymph node metastasis (pN1-). Coverage and intensity of HAS1 immunostainings were recorded from 95 and 96 samples, respectively. Coverage and intensity of HAS2 immunostainings were recorded from 90 samples. Statistically significant differences between the stages are indicated with brackets (Mann–Whitney U test). * *p*-value < 0.05, ** *p*-value <0.01, *** *p*-value <0.001. (TIF 1091 kb)

## Abbreviations

HA: Hyaluronan
HAS: Hyaluronan synthase
HYAL: Hyaluronidase
DSS: Disease specific survival
RFS: Recurrence free survival
LN: Lymph node
PB: Phosphate buffer
BSA: Bovine serum albumin
