# Supplementary figures and images for: Decreased expression of hyaluronan synthase 1 and 2 associates with poor prognosis in cutaneous melanoma
Source: BMC Cancer. 2016 May 16;16:313. doi: 10.1186/s12885-016-2344-8 (PMC4867536; doi:10.1186/s12885-016-2344-8)

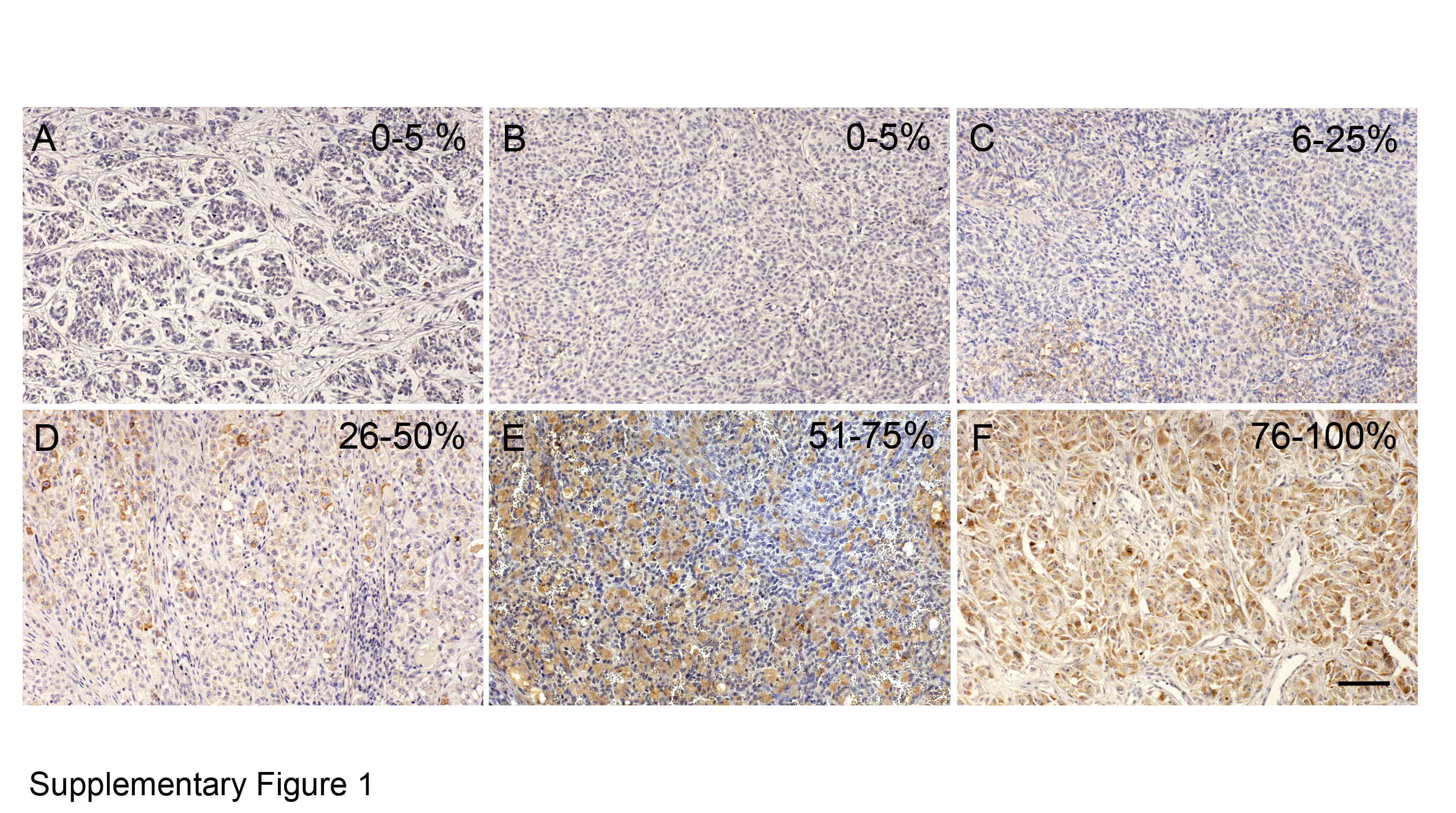

Supplement: Additional file 1: Figure S1. — Evaluation of immunopositivity using five-level scoring system. a and b representing 0-5 % of melanoma cells stained positively, c representing 6-25 % of melanoma cells stained positively, d representing 26-50 % of melanoma cells stained positively, e representing 51-75 % melanoma cells stained positively and f representing 76-100 % of melanoma cells stained positively. Scale bar 100 μm. (TIF 19623 kb) [file 12885_2016_2344_MOESM1_ESM.tif]

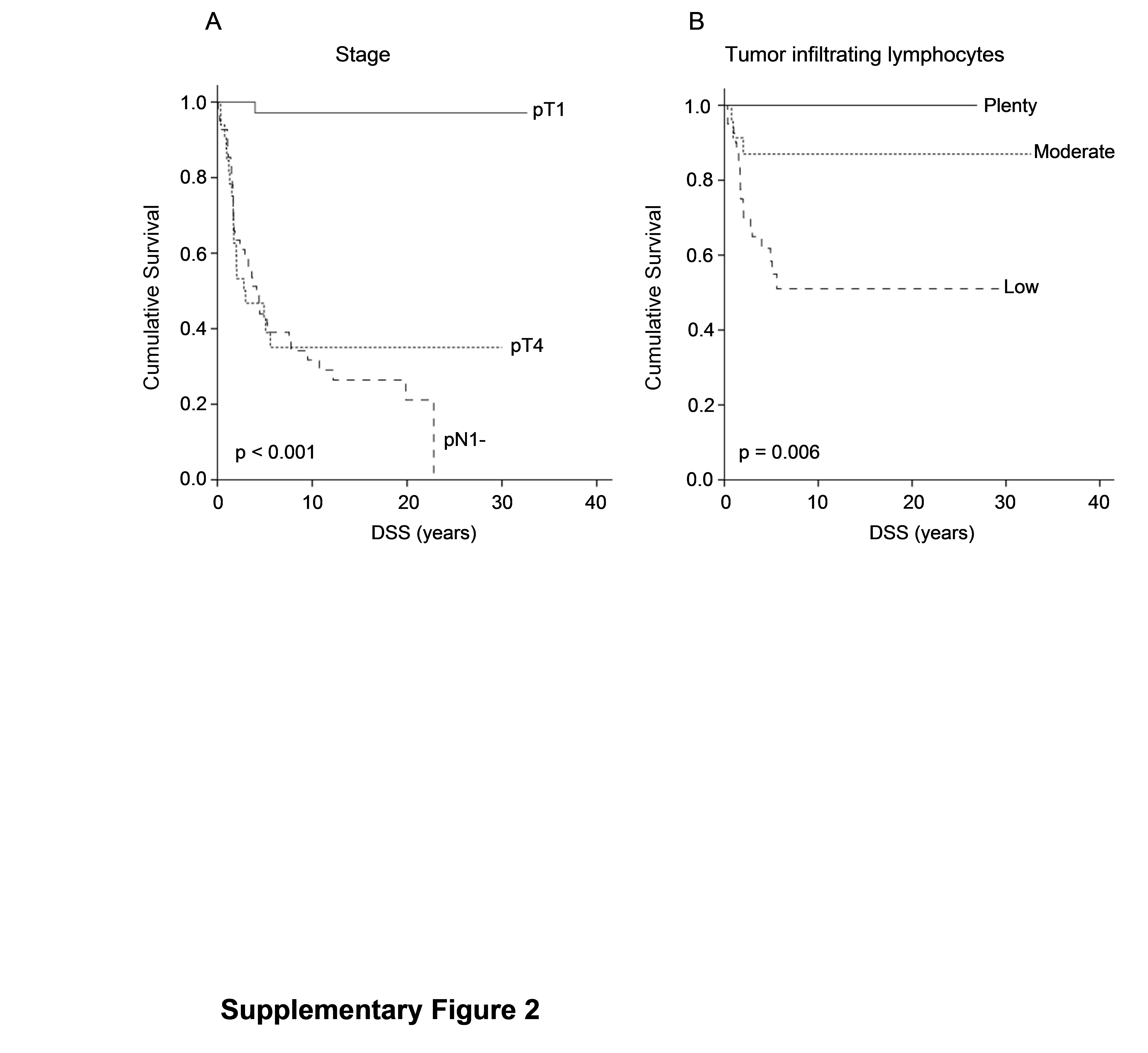

Supplement: Additional file 2: Figure S2. — Clinical data’s accuracy was verified with two Kaplan-Meier log rank tests. Kaplan-Meier log rank test according stage (pT1, pT4 and pN1-) a. Patients with pT1 melanoma had better prognosis than patients with pT4 melanoma or lymph node metastasis (p <0.001). Kaplan-Meier log rank test according tumor-infiltrating lymphocytes –status b. Tumor infiltrating lymphocytes status of pT1 and pT4 melanomas were analyzed and higher amounts of tumor-infiltrating lymphocytes associated with better prognosis (p = 0.006). DSS = disease-specific survival. (TIF 536 kb) [file 12885_2016_2344_MOESM2_ESM.tif]

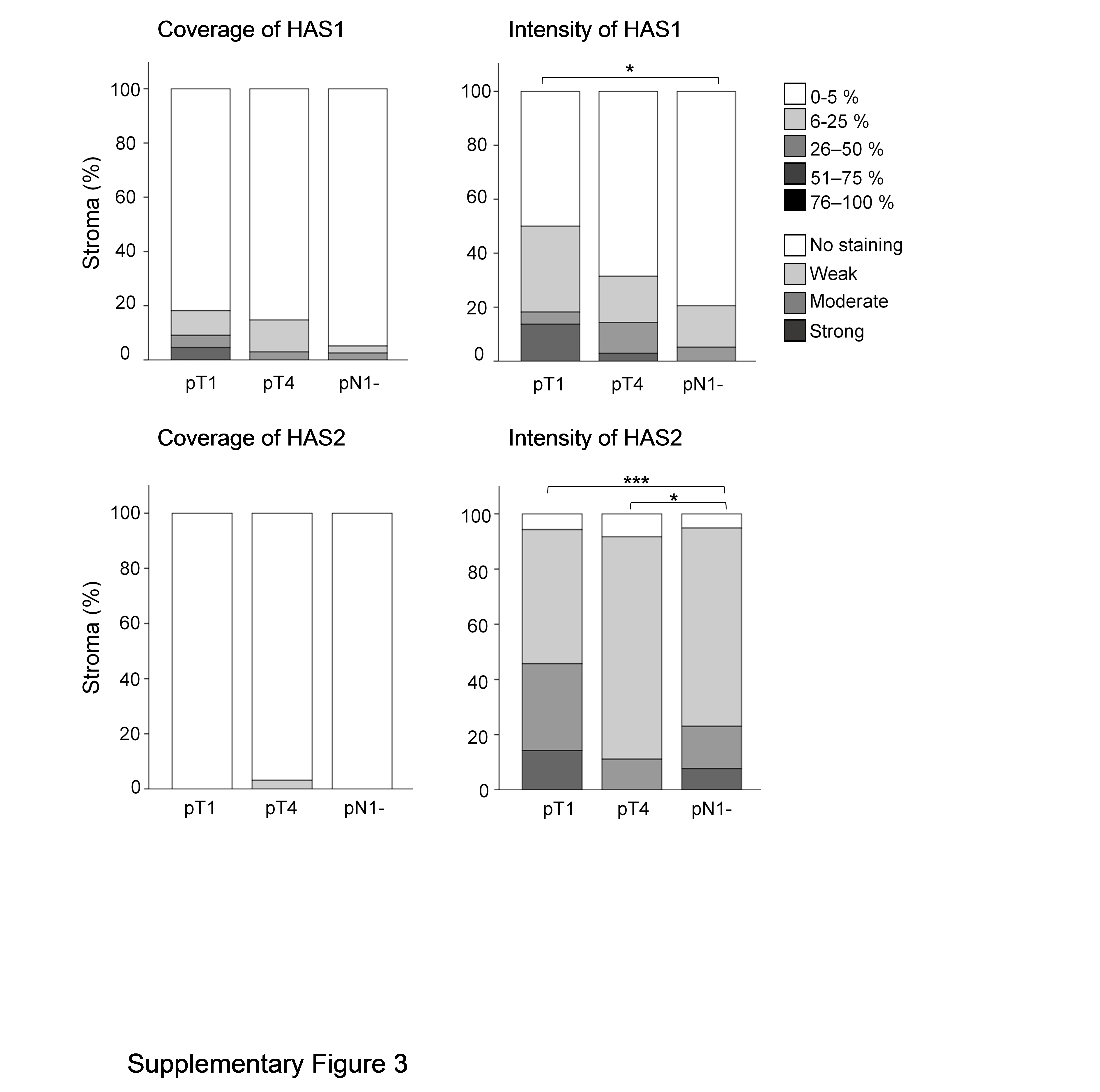

Supplement: Additional file 3: Figure S3. — Immunostaining results of HAS1 and HAS2 in stromal cells. Coverage and intensity of HAS1 and HAS2 immunostainings in the stroma of superficial melanoma (pT1), deep melanoma (pT4) and lymph node metastasis (pN1-). Coverage and intensity of HAS1 immunostainings were recorded from 95 and 96 samples, respectively. Coverage and intensity of HAS2 immunostainings were recorded from 90 samples. Statistically significant differences between the stages are indicated with brackets (Mann–Whitney U test). * p-value < 0.05, ** p-value <0.01, *** p-value <0.001. (TIF 1091 kb) [file 12885_2016_2344_MOESM3_ESM.tif]
